# Early acquired resistance to EGFR-TKIs in lung adenocarcinomas before radiographic advanced identified by CT radiomic delta model based on two central studies

**DOI:** 10.1038/s41598-023-42916-2

**Published:** 2023-09-20

**Authors:** Xiumei Li, Chengxiu Zhang, Tingting Li, Xiuqiang Lin, Dongmei Wu, Guang Yang, Dairong Cao

**Affiliations:** 1 Department of Radiology, The First Affiliated Hospital of Fujian Medical University, 20 Cha-Zhong Road, Fuzhou, 350005 Fujian China; 2 Shanghai Key Laboratory of Magnetic Resonance, School of Physics and Electronic Science, East China Normal University, 3663 North Zhangshan Road, Shanghai, 200062 China; 3 Department of Radiology, The Second Affiliated Hospital of Xiamen Medical College, Xiamen, 361021 Fujian China; 4 Department of Radiology, National Regional Medical Center, Binhai Campus of the First Affiliated Hospital, Fujian Medical University, Fuzhou, 350212 Fujian China; 5 Fujian Key Laboratory of Precision Medicine for Cancer, The First Affiliated Hospital, Fujian Medical University, Fuzhou, 350005 Fujian China; 6 Key Laboratory of Radiation Biology of Fujian Higher Education Institutions, The First Affiliated Hospital, Fujian Medical University, Shanghai, 200062 China

**Keywords:** Medical research, Oncology

## Abstract

Early acquired resistance (EAR) to epidermal growth factor receptor tyrosine kinase inhibitors (EGFR-TKIs) in lung adenocarcinomas before radiographic advance cannot be perceived by the naked eye. This study aimed to discover and validate a CT radiomic model to precisely identify the EAR. Training cohort (n = 67) and internal test cohort (n = 29) were from the First Affiliated Hospital of Fujian Medical University, and external test cohort (n = 29) was from the Second Affiliated Hospital of Xiamen Medical College. Follow-up CT images at three different times of each patient were collected: (1) baseline images before EGFR-TKIs therapy; (2) first follow-up images after EGFR-TKIs therapy (FFT); (3) EAR images, which were the last follow-up images before radiographic advance. The features extracted from FFT and EAR were used to construct the classic radiomic model. The delta features which were calculated by subtracting the baseline from either FFT or EAR were used to construct the delta radiomic model. The classic radiomic model achieved AUC 0.682 and 0.641 in training and internal test cohorts, respectively. The delta radiomic model achieved AUC 0.730 and 0.704 in training and internal test cohorts, respectively. Over the external test cohort, the delta radiomic model achieved AUC 0.661. The decision curve analysis showed that when threshold of the probability of the EAR to the EGFR-TKIs was between 0.3 and 0.82, the proposed model was more benefit than treating all patients. Based on two central studies, the delta radiomic model derived from the follow-up non-enhanced CT images can help clinicians to identify the EAR to EGFR-TKIs in lung adenocarcinomas before radiographic advance and optimize clinical outcomes.

## Introduction

Epidermal growth factor receptor tyrosine kinase inhibitors (EGFR-TKIs) were permitted as the first-line targeted drugs for the treatment of advanced lung adenocarcinoma with mutated EGFR in the guidelines of the national comprehensive cancer network (NCCN)^1^ and the European society of medical oncology (ESMO)^2^ due to its longer progression-free survival than chemotherapy^3^. However, all patients eventually develop into acquired resistance to EGFR-TKIs after an average period of 8 to 16 months^4,5^. The mechanism of drug resistance of first/second-generation EGFR-TKIs in non-small cell lung cancer (NSCLC) is a secondary gene mutation, such as TK domain mutation (T790M), MET amplification, and RAS mutation^6–8^. Substantial evidence has been reported that the secondary gene mutation predates radiographic advance (early acquired resistance) and aggravates tumor heterogeneity^9,10^. Since the size of the tumor in the early acquired resistance period does not increase, the early acquired resistance to EGFR-TKIs before radiographic advance can hardly be perceived by the naked eye of clinicians. Therefore, identifying the patients with early acquired resistance to EGFR-TKIs before radiographic advance is crucial to devising appropriate treatment strategies for optimized clinical outcomes. However, the problem remains extremely challenging.

Radiomic features extracted from medical images can reflect tumor heterogeneity, which could be an expression of underlying genetic alterations^11–13^. Previous studies have reported that radiomics has potential in the prediction of prognosis after chemotherapy^14^, radiotherapy^15^, and immunotherapy^16^. O'Connor and colleagues revealed the effectiveness and necessity of various quantitative imaging biomarkers in the clinical development of targeted therapeutics for the early prediction of clinical outcomes^17,18^.

Follow-up CT tumor images have a fundamental role in response evaluation to EGFR-TKIs. We hypothesized that the hidden information about tumor genetic alterations in the follow-up CT images after EGFR-TKIs therapy can be identified by the image radiomic features. Thus, we aimed to develop and validate a radiomic model to identify the early acquired resistance to EGFR-TKIs in lung adenocarcinomas before radiographic advance. We also aimed to investigate the biological implications of the features in the radiomic model. As far as we know, rare studies have been reported on it.

## Materials and methods

### Studied patient selection

**Figure 1:**
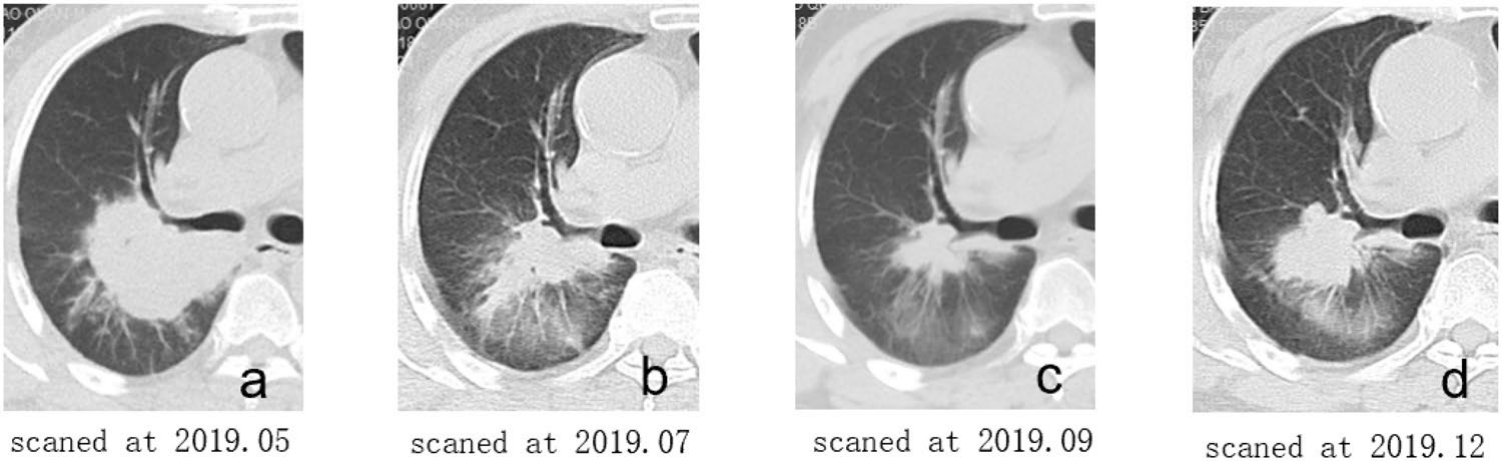
Illustrations of follow-up CT non-enhanced lung images at four different time points. Follow-up CT images of a 66 years old man who underwent EGFR-TKIs therapy because of lung adenocarcinoma with exon 19 deletion mutation. (**a**) the baseline image before TKIs therapy, included in the baseline images; (**b**) the first follow-up image after TKIs therapy, included in FFT; (**c**) the last follow-up image before radiographic advance, included in EAR; (**d**) the radiographic advance image.

This study followed the TRIPOD statement^19^. Institutional review boards of both the First Affiliated Hospital of Fujian Medical University (FAHF) and the Second Affiliated Hospital of Xiamen Medical College (SAHX) approved this retrospective study and waived the requirement for written informed consent. Consecutive patients, who accepted non-surgical treatments for lung carcinoma from December 2016 to May 2022, were enrolled from two institutional databases. The inclusion criteria were as follows: (1) patients with lung adenocarcinoma and confirmed EGFR sensitive mutation of exon 19 deletion mutation (19DEL) and exon 21 L858 point mutation (L858R) by gene detection; (2) only received first/second-generation EGFR-TKIs therapy; (3) had follow-up non-enhanced CT (NCCT) chest scans at four different time points (Fig. 1): baseline images before EGFR-TKIs therapy; first follow-up images after EGFR-TKIs therapy; early acquire resistant images, which were the last follow-up images before radiographic advance; and radiographic advance images. (4) Slice thickness of lung window image ≤ 5 mm. The exclusion criteria were as follows: (1) patients accepted other therapies during EGFR-TKIs therapy; (2) Insufficient image quality due to image artifacts; (3) The margin of the tumor was difficult to delineate.

According to Response Evaluation Criteria In Solid Tumors (RECIST) 1.1^20^, the criteria for acquired resistance to EGFR-TKIs is progressive disease: at least 20% increase in the sum of diameters of all measured target lesions, compared to the smallest sum of diameters of all target lesions recorded at or after baseline (the sum must demonstrate an absolute increase > 5 mm). The follow-up NCCT lung images of each patient scanned at three different times were collected and used to build the models: the baseline images before EGFR-TKIs therapy (Baseline), the first follow-up images after EGFR-TKIs therapy (FFT), and the early acquired resistance (EAR) to EGFR-TKIs, which were the last follow-up images before radiographic advance. Since the aim of our study is to identify the early acquired resistance to EGFR-TKIs in lung adenocarcinomas before the radiographic advance, EAR was marked as positive while the FFT was marked as negative.

### CT acquisition and tumor volume segmentation

CT scans of FAHF cohort were performed on one of the two CT systems (Toshiba: Aquilion CXL 64-slice CT, Aquilion One 320-slice CT). CT scans of SAHX cohort were performed on one of the two CT systems (GE Discovery 64-slice CT, GE Revolution ACTS 16-slice). CT scan parameters were as follows: tube voltage:120 kV; automatic tube current modulation:100–400 mA; rotation time:0.5–1.0 s; Field of view:350 mm × 350 mm; matrix:512 × 512; convolution kernels: B52f.; reconstruction thicknesses and intervals:1.0 mm or 1.25 mm; slice thickness:1.0–5.0 mm (depending on scanners). Interpretation of CT images was done on a lung window (L, –500; W, 1500) by using a workstation on picture archiving and communication system. Delineation of the volume of interest in the targeted tumor was performed with 3D slicer (http://www.slicer.org) by a radiologist with 7 years of experience. The delineation was then reviewed by a radiologist with 15 years of experience in lung cancer diagnosis, who modified the delineation when necessary. All the tumors in the training, internal test, and external test cohorts were segmented manually slice-by-slice. The volume of interest enclosing the CT lung lesion was further refined by excluding areas of fat, air, necrosis, and calcification.

### Radiomic feature extraction and model development

**Figure 2:**
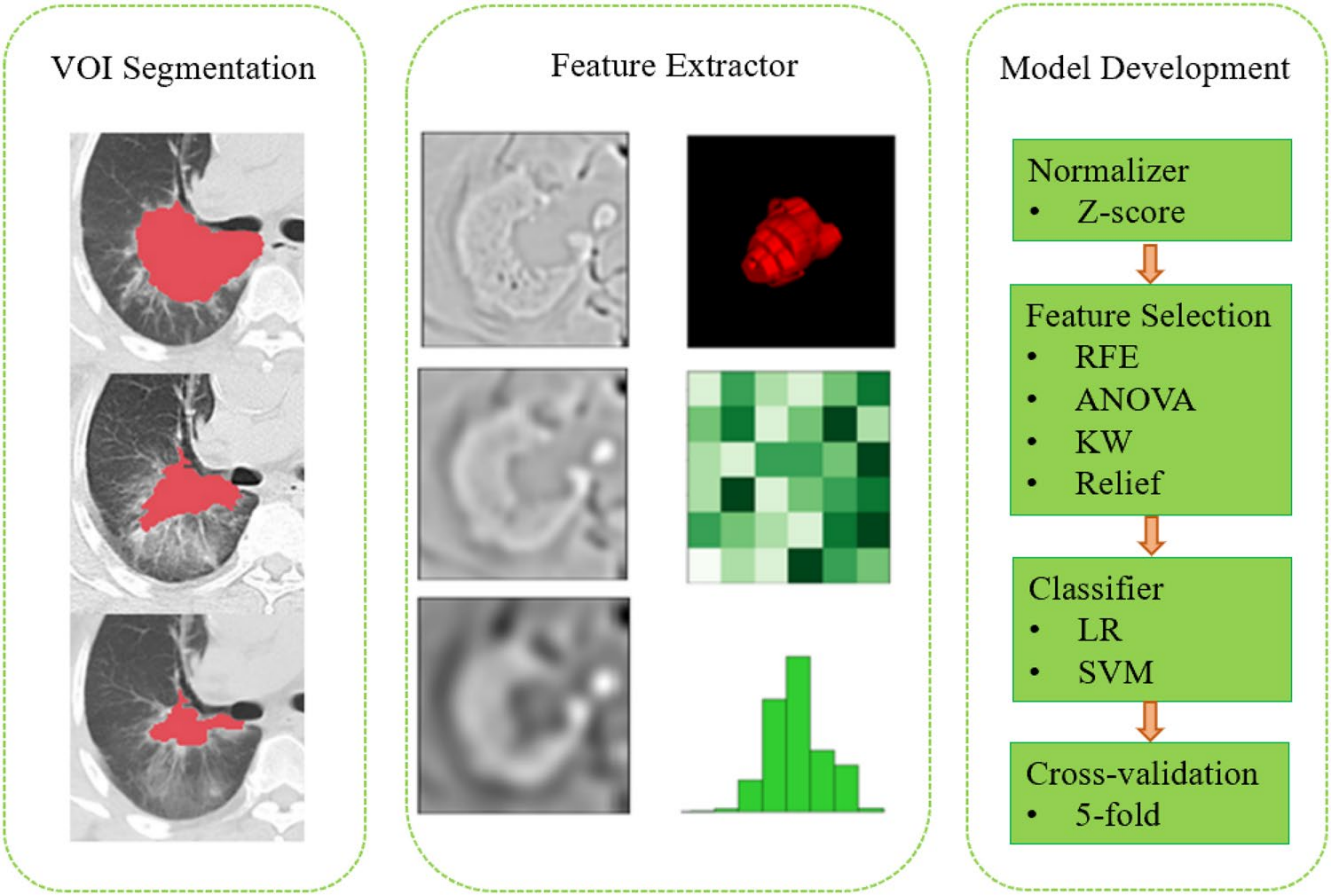
Radiomic analysis workflow.

We resampled all the images to 0.702 mm × 0.702 mm and extracted features using Pyradiomics (Ver. 3.0)^21^ from four base images: original CT image and 3 images filtered by Laplacian of Gaussian (LoG) filters with different Gaussian kernel sizes ($\sigma$ =1.5, 3.0, 5.0). From each of the four base images, we extracted 18 first-order features, 14 shape features, and 73 texture features with a histogram bin count of 50. Texture features included gray level co-occurrence matrix (GLCM), gray level size zone matrix (GLSZM), gray level run length matrix (GLRLM), gray level dependence matrix (GLDM), and neighborhood gray-tone difference matrix (NGTDM). A total number of 378 IBSI (International Biomarker Standardization Initiative)^22^ compliant features were extracted from each lesion for model building. All features were normalized using z-score before further processing. For model development, we used a process similar to previous reported^23^: (1) To reduce the feature dimension and redundancy, for each pair of features whose Pearson Correlation Coefficient (PCC) was larger than 0.98, one random feature was excluded for further model building; (2) For further feature selection and model building, we tried to use different combinations of four feature selection algorithms (Analysis of variance, Kruskal–Wallis, Relief, and Recursive Feature Elimination) and 2 classifiers (Support Vector Machine, and Logistic Regression) to build the classification model with fivefold cross-validation. The workflow of the radiomic analysis is shown in Fig. 2. The above procedure was implemented with open-source software Feature Explorer (version 0.5.2)^24^, which uses scikit-learn (version 0.22.2) as the backend for machine learning. Feature Explorer can semi-automatically try out different combinations of feature selection algorithms and classifiers using specified cross-validation scheme to find the best models according their average cross-validation performance.

### Scout model and final model building

**Figure 3:**
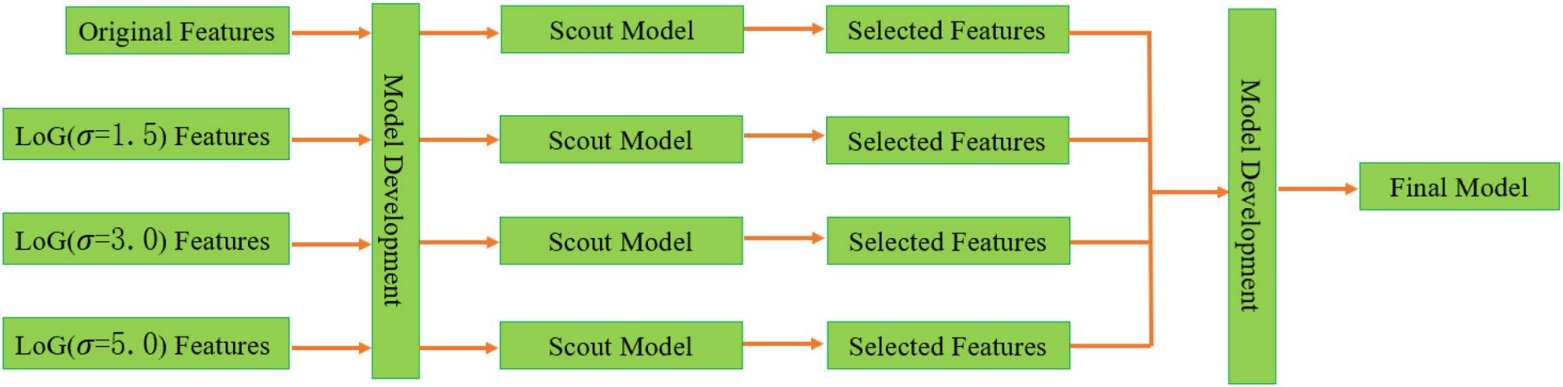
The scout and final radiomic model building process.

Combination of high dimensions and small sample sizes can easily bring about overfitting or the problem of “curse of high-dimensionality”. Therefore, besides the typical dimension reduction and feature selection algorithms, we also used scout model for feature selection, where a model was built for a sub-group of features solely to select useful features in the sub-group^25^. With features extracted from each of the four base images (original CT image and three images filtered by aforementioned different LoG filters), we built a scout model, using fivefold cross-validation over the training dataset. If the average cross-validation AUC ≥ 0.6, all the features retained in the scout model were kept for the final model building. The whole process was shown in Fig. 3.

The building process of the final combined radiomic model was similar to those used for scout models, the only difference is that it used features retained in the scout models, and PCC was not used for feature reduction.

We used features extracted from FFT and EAR images from FAHF cohort to construct a classic radiomic model to identify the early acquired resistance. Furthermore, we used delta features (difference of features between the baseline images and either FFT or EAR from FAHF cohort) to construct the delta radiomic model. The classic radiomic model and delta radiomic model were validated using the data from SAHX cohort.

### Statistical analysis and model evaluation

We used R Studio (version 2022.07.1) with R (version 4.2.1), IBM SPSS Statistics software (version 25.0), SciPy (version 1.4.1), and scikit-learn (version 0.22.2) libraries for statistical analyses. Continuous variables of normal distribution were expressed as mean ± standard deviation and compared by using the Student’s t-test, while continuous variables of non-normal distribution were listed as median (interquartile range) and compared with the Mann–Whitney U test. Categorical variables were expressed as numbers and percentages and compared using the Chi-squared test. Two-sided *p*-values < 0.05 was considered statistically significant. The receiver operator characteristic curve (ROC) analysis was used to evaluate the performance of the model. Besides area under the curve (AUC), the accuracy, sensitivity, specificity, positive predictive value (PPV), and negative predictive value (NPV) were also calculated, using a cut-off value determined by maximizing Youden index over the training cohort. Furthermore, decision curve analysis was also used to evaluate the clinical usability of the model.

### Ethics statement

The studies involving human participants were reviewed and approved by the First Affiliated Hospital of Fujian Medical University and the Second Affiliated Hospital of Xiamen Medical College. Written informed consent for participation was waived for this study due to its retrospective nature. The waiving committees were the First Affiliated Hospital of Fujian Medical University and the Second Affiliated Hospital of Xiamen Medical College.

## Results

### Study cohorts

**Figure 4:**
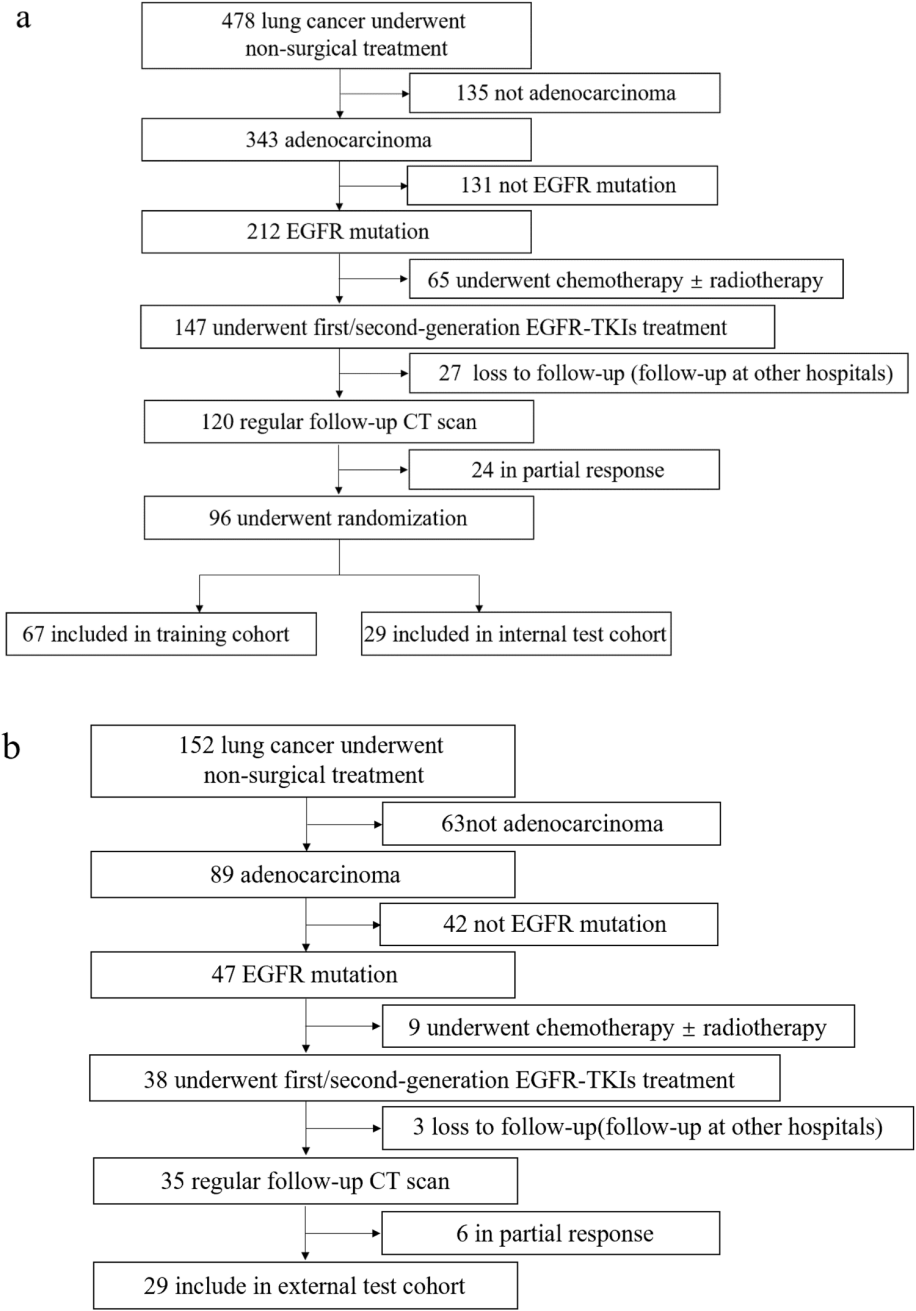
Patient selection flowcharts in (**a**) the First Affiliated Hospital of Fujian Medical University and (**b**) the Second Affiliated Hospital of Xiamen Medical College.

After the choice, 96 patients from the FAHF (Fig. 4a) and 29 patients from the SAHX (Fig. 4b) were included in the study. The FAHF cohort was randomly split into a training cohort (n = 67) and an internal test cohort (n = 29). Random re-splitting was used to ensure that there was no signification difference between the distributions of major clinical characteristics in the training and internal test cohorts (Table 1).

**Table 1:** Clinical characteristics in the training and internal test cohorts.

Characteristics	Training cohort	Internal test cohort	*p*-value
Age (year)	62.9 ± 9.5	60.3 ± 11.2	0.239*
Gender (M/F)	30/37	13/16	0.996 #
Smoking (0/1)	59/8	23/6	0.265 #
Location (1/2)	8/59	5/24	0.486#
Max Diameter	4.0 ± 1.7	3.9 ± 1.5	0.784*
Shrinkage Rate	0.327 ± 0.171	0.349 ± 0.155	0.555*
Response Days	301.1(137.0,343.0)	317.1(188.0,339.5)	0.949&

^#^Chi-square test, *Student 's test, & Mann–Whitney test.

Max Diameter is the maximum diameter in the maximum transverse section of lung adenocarcinoma. Shrinkage Rate is calculated by subtracting the maximum diameter in the baseline image from maximum diameter in the FFT then divided by the maximum diameter in the baseline image. Response Days are the days between the baseline scan and early acquire resistance scan. Location 1 and 2 denote central and peripheral types of lung cancer, respectively.

### Radiomic model

**Figure 5:**
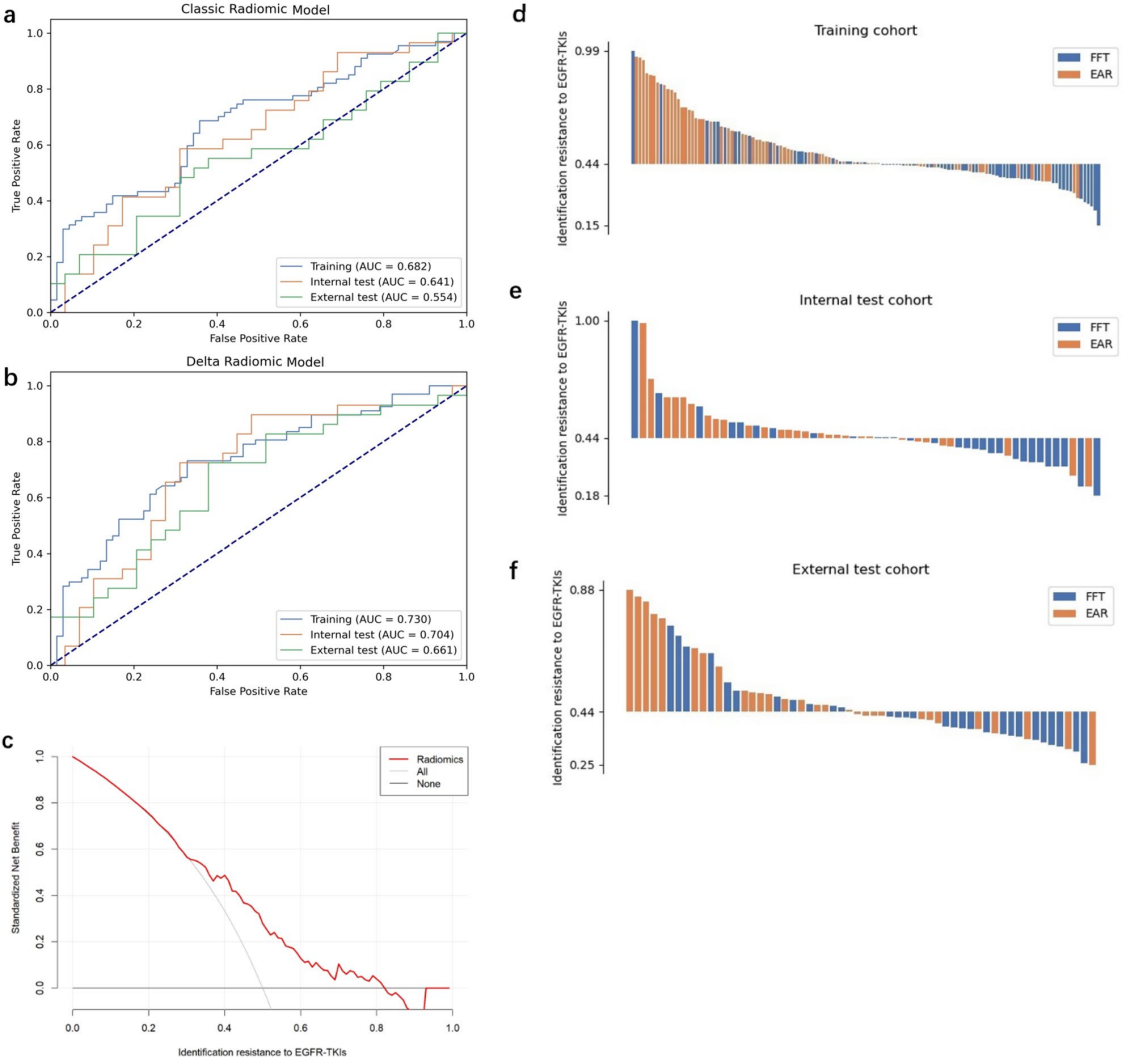
Comparison of different models for identifying early acquired resistance in training, internal test, and external test cohorts. (**a**) and (**b**) are the ROC curves of the classic radiomic model and delta radiomic model over different cohorts, respectively. The delta radiomic model showed better performance. (**c**) Decision curve analysis was performed for the delta radiomic model in all patients from two institutions, which indicated that the delta model added more benefit than treating all or none of the patients for the range of 0.3 to 0.82 threshold probabilities. (**d**-**f**) Waterfall plot of the combined model showing the predicted probabilities in training, internal and external test cohorts, respectively.

For the classic radiomic model, the average cross-validation AUC values of the 4 scout models were 0.623, 0.647, 0.655, and 0.601, respectively. Features retained in 4 models were 6, 3, 1, and 14, respectively. Details of the scout models are summarized in Table 2. All 24 retained features were combined to build the final model. The combination of Kruskal–Wallis (KW) for feature selection and Logistic Regression (LR) for classifier yielded the model with best performance. The coefficients of 7 features used in the model are listed in Table 3. It achieved AUC values of 0.682 (95% CI: 0.591–0.772), 0.641 (95% CI: 0.492–0.786) and 0.554 (95% CI: 0.399–0.708) over the training, internal test and external test cohorts, respectively. See Table 4 for detailed metrics. The ROC curves are shown in Fig. 5a.

**Table 2:** Details of scout models.

Model	Base image	Feature selection		Average CV AUC			Feature number	Features
Classic	Original	ANOVA LR		0.623			6	glcm_Contrast, glcm_DifferenceVariance, ngtdm_Contrast, shape_MajorAxisLength, shape_MinorAxisLength, shape_SurfaceVolumeRatio
	LoG (σ = 1.5)	KW LR		0.647			3	firstorder_10Percentile, firstorder_Mean, firstorder_Variance
	LoG (σ = 3)	ANOVA LR		0.655			1	glcm_DifferenceEntropy
	LoG (σ = 5)	Relief LR		0.601			14	glrlm_LongRunHighGrayLevelEmphasis, firstorder_RootMeanSquared, firstorder_90Percentile, glszm_SmallAreaLowGrayLevelEmphasis, glcm_SumEntropy, firstorder_Maximum, glcm_ClusterShade, gldm_LargeDependenceHighGrayLevelEmphasis, glszm_LargeAreaLowGrayLevelEmphasis, glszm_LargeAreaEmphasis, glszm_HighGrayLevelZoneEmphasis, firstorder_InterquartileRange, glrlm_GrayLevelNonUniformityNormalized, glrlm_LongRunEmphasis
Delta	Original	ANOVA LR		0.651			6	glcm_Contrast, glcm_DifferenceVariance, glszm_LowGrayLevelZoneEmphasis, ngtdm_Coarseness, ngtdm_Contrast, shape_SurfaceVolumeRatio
	LoG (σ = 1.5)	ANOVA SVM		0.677			4	firstorder_10Percentile, firstorder_Mean, firstorder_Median, ngtdm_Strength
	LoG (σ = 3)	KW LR		0.719			1	glcm_DifferenceVariance
	LoG (σ = 5)	KW LR		0.639			4	glcm_Idmn, glcm_Idn, glcm_InverseVariance, ngtdm_Contrast

^#^LoG: Laplacian of Gaussian, LR: Logistic Regression, KW: Kruskal–Wallis, SVM: Support Vector Machine, CV: Cross-validation.

**Table 3:** Coefficients of features in the radiomic models.

Model	Features	Coefficients	Intercept
Classic	log-sigma-3–0-mm-3D_glcm_DifferenceEntropy	0.738	0.0173
	original_glcm_DifferenceVariance	− 0.016	
	log-sigma-1–5-mm-3D_firstorder_10Percentile	− 0.038	
	log-sigma-1–5-mm-3D_firstorder_Mean	− 0.318	
	log-sigma-1–5-mm-3D_firstorder_Variance	0.179	
	log-sigma-5–0-mm-3D_glszm_LargeAreaEmphasis	− 0.442	
	log-sigma-5–0-mm-3D_glrlm_LongRunEmphasis	0.804	
Delta	log-sigma-3–0-mm-3D_glcm_DifferenceVariance	0.90483	0.06478

**Table 4:** Detailed performance metrics of the classic radiomic model.

Cohort	AUC	95% CI	Accuracy	Sensitivity	Specificity	PPV	NPV
Training	0.682	0.591–0.772	0.664	0.687	0.642	0.657	0.672
Internal test	0.641	0.492–0.786	0.638	0.586	0.690	0.654	0.625
External test	0.554	0.399–0.708	0.586	0.483	0.690	0.609	0.571

^#^Cut-off value: 0.4542.

For the delta radiomic model, the average cross-validation AUC values of the 4 scout models were 0.651, 0.677, 0.719, and 0.639, respectively. Features retained in the 4 models were 6, 4, 1, and 4, respectively (Table 2). All 15 retained features were combined to build the final delta radiomic model. The best final model used KW for feature selection and LR for classifier and contained 1 feature, namely LoG-sigma-3–0-mm-glcm_DifferenceVariance. It achieved AUC values of 0.730 (95% CI: 0.646–0.815), 0.704 (95% CI: 0.564–0.845), and 0.661 (95% CI: 0.519–0.806) over the training, internal test, and external test cohorts, respectively. The detailed metrics are listed in Table 5. ROC curves of the model over the training, internal test, and external test cohorts are shown in Fig. 5b and the decision curve is shown in Fig. 5c. The decision curve shows that when the threshold of the probability of the initial acquire resistance to EGFR-TKIs was between 0.3 and 0.82, the target identification adds more benefit than treating all patients. Waterfall plots of the combined model showing the predicted probabilities in training, internal and external test cohort are shown in Fig. 5d–f.

**Table 5:** Detailed performance metrics of the delta radiomic model.

Cohort	AUC	95% CI	Accuracy	Sensitivity	Specificity	PPV	NPV
Training	0.730	0.646–0.815	0.701	0.731	0.672	0.690	0.730
Internal test	0.704	0.564–0.845	0.707	0.724	0.690	0.700	0.704
External test	0.661	0.519–0.806	0.672	0.724	0.621	0.656	0.692

^#^Cut-off value: 0.4438.

## Discussion

Despite the success of EGFR-TKIs therapy in the treatment of lung cancer, the acquired resistance limits the ability to translate this method into a curative treatment. The mechanisms of acquired resistance have traditionally been thought of as genetic alterations, which can be associated with tumor heterogeneity and hypoxia^26–28^. Tumor heterogeneity and hypoxia, which can increase cellular resistance to chemotherapy, radiotherapy, and inhibition of immune responses, may not be perceptible to the naked eye but can be quantified by using texture analysis^29–31^. Therefore, we applied radiomics to follow-up CT images to identify the patients with early acquired resistance to EGFR-TKIs before radiography advance. However, in our study, the classic radiomic model failed to achieve satisfactory performance in identifying the early acquired resistance. There are likely multiple mechanisms responsible for this finding. First, the number of early acquired resistance cells (i.e., the secondary gene mutation cells) may make up only a small proportion of tumors in EAR images^32^. Second, the classic radiomic model did not have the capacity to distinguish the little difference in radiomic features between the FFT and EAR because both the FFT and EAR belong to the progression-free survival stage. Although the classic radiomic model barely worked, it was no worse than the naked eye, which cannot identify EAR either, because the size of the tumor in EAR does not increase.

Recently, there has been an increasing interest in delta-radiomics^33,34^, which calculates the changes of radiomic features extracted from the dynamic follow-up treatment images, such as pre-therapy and post-therapy images. Delta-radiomics can be more sensitive to changes in texture or homogeneity over the period, which was also demonstrated by our results. In our study, delta radiomic model achieved better performance than the classic radiomic model (internal test AUC: 0.704 vs 0.641, external test AUC: 0.661 vs 0.554). Our results showed that the difference in radiomic features between pre-therapy and post-therapy can reflect the changes of intra-tumor heterogeneity and hypoxia, which can be used to identify EAR.

Our final delta radiomic model only contained one feature, namely, LoG-sigma-3–0-mm-GLCM_DifferenceVariance. This feature was positively associated with the degree of drug resistance. According to the Image Biomarker Standardization Initiative (IBSI)^22^, GLCM is a matrix to express how combinations of discretized intensities (grey levels) of neighboring pixels or voxels in a 3D volume. Meanwhile, GLCM is distributed along one of the image directions. High GLCM might be more representative of heterogeneous, such as chaotic vascularization^35,36^. Yu et al. investigated the association between radiomic features extracted from diagnostic CT images and clinical outcomes in Stage I non-small cell lung cancer in a single institution^37^. They stated that GLCM showed a strong positive correlation with the mortality risk index (Spearman correlation coefficient 0.86, *P* < 0.001). Similarly, Tunali et al. demonstrated that GLCM inverse difference was positively associated with tumor hypoxia, tumor acidosis, and treatment resistance^38^. Our findings were consistent with those previous studies, indicating that the GLCM features were correlated to heterogeneity of the tumor, which in turn was associated with the tumor resistance to EGFR-TKIs.

If this delta radiomic model for EAR prediction can be further refined with more data, it can potentially be used when the routine follow-up CT examination after EGFR-TKIs therapy is taken. It can tell the clinicians the risk of EAR of the patient, without any extra expenditures and radiation exposures. It can provide a convenient, non-invasive and personalized approach to predict whether drug resistance has happened, thus can help clinicians to change the drug used or make other therapeutic decisions. It is conducive to precision medicine and improves prognosis.

We acknowledge that our study did have its limitations. First, due to the retrospective design of our study, selection bias was inevitable. Second, the slice thickness of CT images was different (1–5 mm) caused by differences CT imaging parameters from the two hospitals, which might have negative influences on the stability of radiomic features. Third, the sample size was relatively small which would affect the robustness of the prediction model, which could be the major reason for the suboptimal performance of the proposed model. A multi-center prospective study involving a more homogenous scan protocol and relevant clinical variables could be performed to further validate and improve the model.

## Conclusion

The delta radiomic model derived from follow-up non-enhanced CT images has the potential to provide a novel, reliable, real-time, and non-invasive detection of early acquired resistance to EGFR-TKI in patients with lung adenocarcinomas. Early detection of acquired resistance may facilitate early adjustment of treatment strategies and can prolong patients' progression-free survival and improve prognosis.

## Data Availability

The datasets used in the present study are available from the corresponding author on reasonable request.
